# In-silico analysis reveals druggable single nucleotide polymorphisms in angiotensin 1 converting enzyme involved in the onset of blood pressure

**DOI:** 10.1186/s13104-021-05879-z

**Published:** 2021-12-20

**Authors:** Brenda Udosen, Opeyemi Soremekun, Chinwe Ekenna, Olaposi Idowu Omotuyi, Tinashe Chikowore, Oyekanmi Nashiru, Segun Fatumo

**Affiliations:** 1 The African Computational Genomics (TACG) Research Group, MRC/UVRI, and LSHTM, Entebbe, Uganda; 2 The African Center of Excellence in Bioinformatics of Bamako (ACE-B), University of Sciences, Techniques and Technologies of Bamako, Bamako, Mali; 3 University at Albany, Albany, NY USA; 4 Department of Biochemistry, Adekunle Ajasin University, Akungba-Akoko, Ondo State, Nigeria; 5 H3Africa Bioinformatics Network (H3ABioNet) Node, Centre for Genomics Research and Innovation, NABDA/FMST, Abuja, Nigeria; 6 Sydney Brenner Institute for Molecular Bioscience, Faculty of Health Sciences, University of the Witwatersrand, Johannesburg, South Africa; 7 MRC/Wits Developmental Pathways for Health Research Unit, Department of Pediatrics, Faculty of Health Sciences, University of the Witwatersrand, Johannesburg, South Africa; 8 Department of Non-Communicable Disease Epidemiology, London School of Hygiene and Tropical Medicine, London, UK

**Keywords:** SNP informatics, Angiotensin-Converting enzyme 1, Single-nucleotide polymorphisms, Blood Pressure

## Abstract

**Objective:**

The Angiotensin 1 converting enzyme (*ACE1*) gene plays a critical role in regulating blood pressure and thus, it has become a major therapeutic target of antihypertensives. Single nucleotide polymorphisms (SNPs) occurring within a gene most especially at the functional segment of the genes alter the structure–function relationship of that gene.

**Results:**

Our study revealed that five nsSNPs of the *ACE1* gene were found to be potentially deleterious and damaging and they include rs2229839, rs14507892, rs12709442, and rs4977 at point mutations P351R, R953Q, I1018T, F1051V, and T1187M. The protein stability predictive tools revealed that all the nsSNPs decreased stability of the protein and the Consurf server which estimates the evolutionary conservation profile of a protein showed that three mutants were in the highly conserved region. In conclusion, this study predicted potential druggable deleterious mutants that can be further explored to understand the pathological basis of cardiovascular disease.

**Supplementary Information:**

The online version contains supplementary material available at 10.1186/s13104-021-05879-z.

## Introduction

Hypertension is a significant health problem worldwide which accounts for an estimated 7.5 million deaths [1]. The *ACE1* gene is a significant component of the renin-angiotensin system (RAS) [2, 3] which helps to regulate blood pressure and converts the hormone angiotensin I to the active vasoconstrictor angiotensin II [4]. The *ACE1* gene is 21 kb in length on the long arm of chromosome 17 (17q23.3) and is made up of 26 exons and 25 introns. Rigat et al*.,* who first reported the *ACE1* gene polymorphism, proposed that this gene's insertion/deletion polymorphic form accounts for half (47%) of the phenotypic variance for serum enzyme level.

Considering the implication of *ACE1* Single nucleotide polymorphisms (SNPs) on the phenotypic variance for serum enzyme level and considering the role this gene plays in high blood pressure, it is necessary to study the implications of its SNPs. Therefore, this study aims to identify deleterious and disease-causing nsSNPs in *ACE1* that could serve as molecular and genetic biomarkers to diagnose high blood pressure and are targeted explicitly by inhibitors.

## Main text

### Materials and methods

#### Single nucleotide polymorphism data retrieval

Non-synonymous SNPs of our target gene were retrieved from the SNPs database (dbSNPs) server of the National Centre for Biotechnology Information (NCBI) [8]. The basis of selection was focused on polymorphisms with small-scale multi-base deletions or insertions and single-base nucleotide substitutions.

#### Phenotype prediction of deleterious nsSNPs

To identify potential deleterious nsSNPs associated with *ACE1* gene of high blood pressure, we used six different bioinformatics tools implemented in the following web servers: the *Sorting Intolerant from Tolerant* (SIFT) [9], *Pro*tein *V*ariation *E*ffect *An*alyzer (PROVEAN) [10], Polymorphism Phenotyping (PolyPhen-2) [11], SNPs&GO [12], Predictor of human Deleterious Single Nucleotide Polymorphism (PhD-SNPs) [13], PANTHER [14]. SIFT server classifies nsSNPs based on tolerance index (TI) to be either tolerated (TI ≥ 0.05) or deleterious (≤ 0.05). PolyPhen-2 classifies nsSNPs as either being possibly/probably (0.00–0.99) damaging or probably benign (≥ 2) by assigning position-specific independent counts (PSIC) score (0 ≤ 2 ≤ *X*) [15]. The SNP with the highest deleterious prediction by at least five in silico tools were considered the most deleterious nsSNPs for *ACE1* and selected for further investigation.

#### Protein stability analysis of predicted *ACE1* nsSNPs

To have a higher prediction accuracy of protein stability changes upon single AA mutation, we used the istable 2.0 server [16] to exploit in-built sequence-based tools like the MUpro [17], interpretable decision tree method iPTREE-STAB [18], I-Mutant 2.0 [19], and also the impact of non-synonymous variations on Protein Stability (INPS) [20]. The I-Mutant tool uses a reliability index (RI) score ranging from 0 through 10 for prediction. DDG is a parameter used by various tools to evaluate the stability of the protein upon mutation at pH 7.0 and 25°c temperatures. A decrease of free energy change (ΔΔG) in the value is encoded as 0, and an increase of free energy change (DDG) is encoded as 1.

#### Protein conservation analysis

To identify putative functional and structural amino acids and estimate their evolutionary conservation profile, we used Consurf [21], a web server tool that uses the Bayesian approach to analyze the evolutionary pattern of the amino acid. The conservation grades were mapped onto the query structure and specified using the Consurf color-code, with cyan-through-purple corresponding to grades 1 (most evolving) through 9 (most evolutionary conserved).

#### Protein modeling of wild and mutant type *ACE1* and structural difference

To predict our protein's three-dimensional (3D) structure and further analyze the difference between the mutant and wild type of *ACE1* protein, we used the homology modeling tool in Robetta [22], the resultant structure was then viewed using Chimera 1.11[23]. Validation of the predicted protein structure was assessed using ERRAT [24], Verify-3D [25], and PROCHECK [26] programs available from the structural and verification analysis server SAVE (http://nihserver.mbi.ucla.edu/SAVES). A TM-align algorithm was then used to compare the wild and the mutant type protein structure of the *ACE1* gene [27].

Molecular docking was carried out using AutoDock vina tools in-built in Chimera (ref). A grid box with coordinate (Centre: x = 17.588, y = 60.433, z = 40.395 and size: x = 23, y = 34, z = 33) was set around the binding site of ACE1 protein to accommodate Benazepril. Benazepril is an inhibitor drug that is used to treat high blood pressure by lowering blood pressure through inhibiting the formation of angiotensin. The 2D structure of Benazepril in mol2 format was retrieved from DrugBank [29], the structure was further optimized using the GAFF forcefield and steepest descent in Avogadro [30].

## Results

### Non-synonymous single nucleotide polymorphism retrieved from dbSNPs database

The identification of disease-causing SNPs is vital to understand the role a protein plays in disease. A total of 80 nsSNPs were retrieved from the National Centre for biotechnology informatics dbSNPs database server [8] (Additional file 1: Table S1). The retrieval process was filtered to retrieve only nsSNPs that had a clinical consequence reported by Clinvar and implicated in High blood pressure [31].

### Identification of deleterious nsSNPs in *ACE1*

All eighty nsSNPs retrieved were subjected to six tools which predicted fifty-nine nsSNPs to be potentially deleterious (Additional file 1: Table S2a). SIFT tool predicted twenty-three nsSNPs to be deleterious (Additional file 1: Table S2b). SNPs&GO tool predicted thirty-seven nsSNPs to be diseased (Additional file 1: Table S2c) while PhD-SNP predicted forty nsSNPs to be diseased (Additional file 1: Table S2c). PANTHER revealed eleven nsSNPs to be diseased (Additional file 1: Table S2c). According to PROVEAN results, thirty-nine nsSNPs were predicted to be deleterious (Additional file 1: Table S2d). Unlike other tools, PolyPhen-2 had the least deleterious prediction of only six nsSNPs (Additional file 1: Table S2e). Fifty-nine SNPs identified to be deleterious across the six tools were shortlisted to five, which included SNPs that were common in at least five of the predictive tools so that only the highly deleterious SNPs will be used for downstream analyses (Table 1).

**Table 1:** Clinically significant information of Deleterious predicted SNPs

SNP ID	Chr17 (GRCh37) location	Nucleotide change	Protein ID	Amino acid change	Functional Consequence
rs2229839	61559033	C > G	ENSP00000290866	P351R	Missense variant
rs143507892	61568688	G > A	ENSP00000290866	R953Q	Coding sequence variant
rs4976	61570937	T > C	ENSP00000290866	I1018T	Coding sequence variant
rs4977	61571297	T > G	ENSP00000397593	F1051V	Coding sequence variant
rs12709442	61574215	C > T	ENSP00000290866	T1187M	Coding sequence variant

### Stability profile prediction of nsSNPs protein in *ACE1*

To predict the protein stability changes, we used the istable algorithm tool, which incorporated results from three sequence-based tools; MUpro, iPTREE-STAB, I-Mutant2.0, and an additional tool, INPS. The five highly deleterious nsSNPs identified from the previous analysis were used to predict protein stability. The five predictive tools revealed that all these nsSNPs decrease stability of *ACE1* protein (Additional file 1: Table S3a–d).

### Conservation prediction of deleterious nsSNPs in *ACE1*

To further explore the possible effect of the five nsSNPs, Consurf was used to reveal the essential functional and structural regions by analyzing the evolutionary pattern of the five nsSNPs protein. The results predicted I1018T and F1051V as structural residues making them highly conserved and buried, while P351R, R953Q, and T1187M were indicated as functional residues making them highly conserved and exposed (Additional file 1: Table S4).

### Comparative *ACE1* modeling of wild and mutant type and structural characterization

**Fig. 1:**
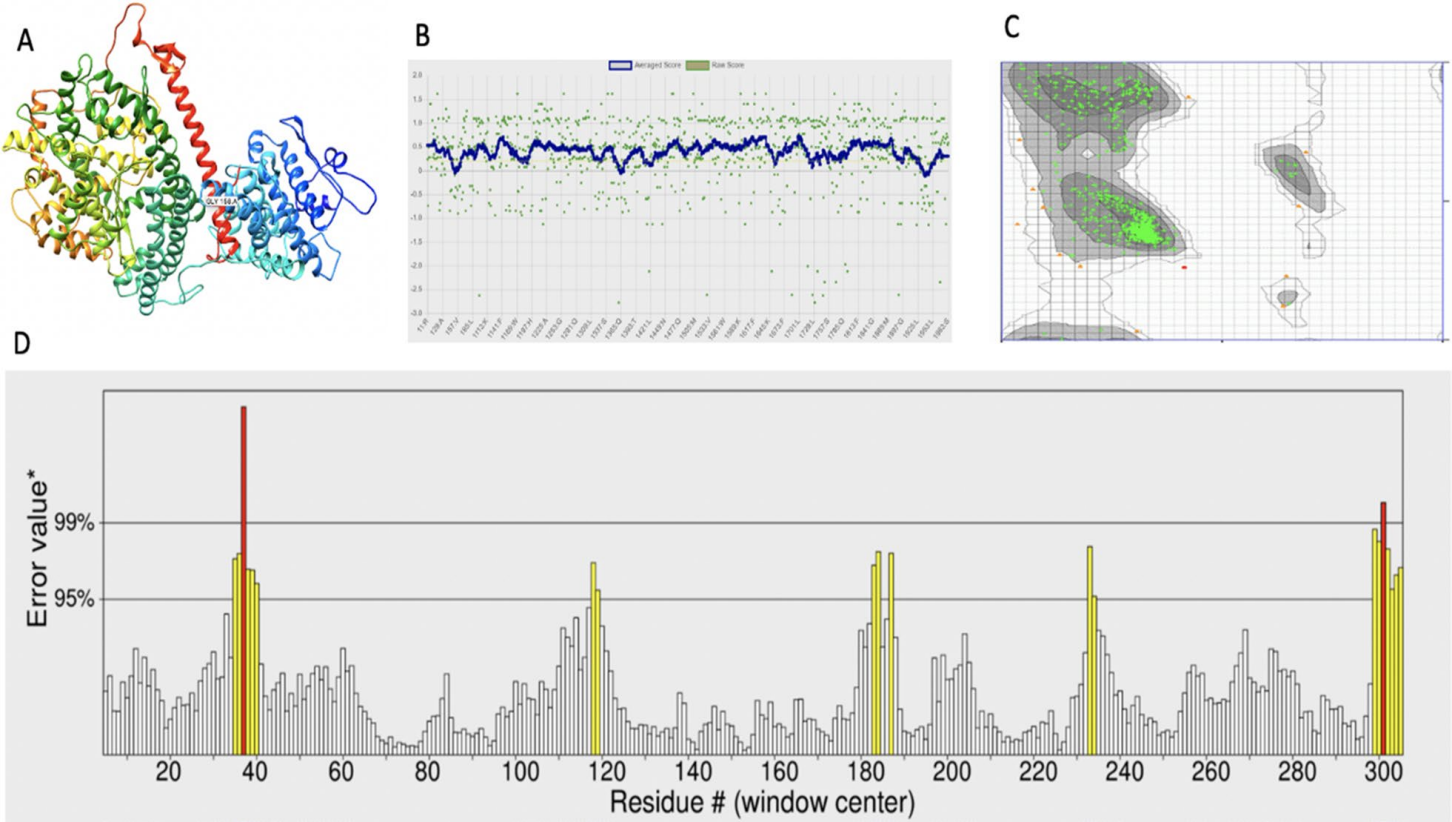
**a** In-silico 3-Dimensional structure of *ACE1* modeled using *ab-initio* homology modeling. **b** Verify-3D plot showed that 89.73% of the residues have averaged a 3D-1D score of ≥ 0.2. **c** Assessment and validation of HBB Protein showing Ramachandra plot obtained by PROCHECK: 92.6% residues in favorable regions; 6.8% residues in additional allowed regions; 0.5% residues in generously allowed regions; 0.1% residues in disallowed regions. **d** ERRAT plot showing 96% quality factor.

The protein sequence of *ACE1* (1300 AA residues) with accession number P12821 was retrieved from the UniProt database [32]. The sequence was then inputted into Robetta [22] and was used as a template to model the 3D structure of *ACE1* (Fig. 1a). Following the validation of the modeled structure in PROCHECK, ERRAT, and Verify-3D [24–26], the output result from Verify-3D revealed that 89.73% of the residues have an average 3D-1D score of ≥ 0.2 [33] (Fig. 1b). The quality of the 3D protein structure was further assessed through the Ramachandran plot available in PROCHECK. The plot from the predictive model showed that 92.6%, 6.8%, 0.5%, and 0.1% residues be in favored, allowed, outlier regions, and disallowed, respectively, confirming that the protein structure is of good quality (Fig. 1c). ERRAT showed a quality factor of 96.395 (Fig. 1d). Generally, results obtained from the above tools suggested that our modeled protein structure is of good quality and thus could be used for downstream analysis.

### ACE1 Mutant type as a potential drug target

To predict the bound conformations and the binding affinity of the molecule with the protein, we performed molecular docking using the AutoDock tool in Chimera [33]. The binding affinity and interacting amino acids residues within the *ACE1* binding pocket of each mutant protein upon binding to Benazepril are highlighted in (Table 2). Some of the interactions exhibited by Benazepril include hydrogen bond, pi-alkyl, pi-pi stacked, etc. (Additional file 2: Figs. S1 and S2).

**Table 2:** Molecular docking of amino acid change against Benazepril

Amino acid change	Interacting amino acids	Binding score (Kcal/mol)
P351R	Tyr805, Cys652, Ala636, Glu658, His635	− 8.7
R953Q	Tyr344, Asn352, Tyr642, Ala345, His692, Ala638, Val800, Trp639	− 9.1
I1018T	Asn348, Asn352, Tyr642, Ala345, Ala638, His692, Val800, Trp639	− 9.0
F1051V	Tyr344, Asn352, Tyr642, Ala345, His692, Val800, Trp639	− 9.2
T1187M	Asn352, Tyr642, Ala345, His692, Ala638, Ser637, Val800, Trp639	− 9.2

## Discussion

We found a total of fifty-nine deleterious nsSNPs, five of which were consistent across the six tools used at mutation points in P351R, R953Q, T1187M, I1018T, and F1051V. The protein stability analysis highlighted five nsSNPs to decreased stability. According to previous studies, decreased protein stability leads to misfolding and degradation of the protein [34]. In addition, using Consurf for conservation analysis showed that three of the amino acid mutation point were highly conserved with a conservation score of 9.

Amino acids involved in protein–protein interaction located at the enzymatic sites are known to be more conserved than others and are also known to be involved in various cellular processes in a biological system including the stability of the genome [35]. For this reason, highly conserved nsSNPs located in the conserved region are more deleterious than nsSNPs located in the variable regions because they destabilize the protein structure and function.

Although the use of a single computational tool cannot generate a substantial predictive result of the functional protein region [36], the implementation of several tools could help provide further insight into the impact of nsSNPs on a protein function. Hence, this current study applied multiple in silico tools including INSP, SIFT, Polyphen2, PhD-SNP, PROVEAN, I-Mutant2.0, MUpro, SNPs&GO, Verify-3D, PROCHECK, ERRAT, and Consurf to identify and evaluate deleterious nsSNPs in the *ACE1* gene. Each of these tools implemented different machine learning approaches such as neural network (NN), decision tree, support vector machine (SVM), and the Bayesian Network to identify nsSNPs that could be used as a drug target for the treatment of high blood pressure [18, 37–39].

## Conclusions

Our results showed that SNPs identified through in-silico analysis can alter the structure and function of the *ACE*1 gene protein. The five nsSNPs analyzed in this study occur in the functional region of the *ACE*1 gene and may therefore altar the functionality of ACE1. This can subsequently be used as a basis for enhancing effective drug discovery and pathogenesis targeting ACE1. One major strength of this study is that it engages different tools which leverage different algorithms for prediction. This further increases the reliability of the predictions.

## Limitation

A major limitation of this study like other in-silico studies is that all the steps taken to predict the impact of the nsSNPs are computer-based, hence, there is a need to explore more robust in-vitro and in-vivo investigations to confirm these result.

## Supplementary Information

**Additional file 1: Table S1.** Nonsynonymous Single Nucleotide Polymorphism of ACE1 gene retrieved from NCBI dbSNP database. **Table S2. a** Deleterious or disease-causing SNPs across the six in silico tools (SNPs&GO, PROVEAN, PhD-SNP, Polyphen-2, PANTHER and SIFT). **b** SIFT predictive result of deleterious of nsSNPs. **c** SNPs&GO deleterious nsSNPs prediction. **d** PROVEAN predictive result of deleterious or disease-causing nsSNPs. **e** Polyphen-2 predictive result of deleterious or disease-causing nsSNPs. **Table S3. a** INSP protein stability predictions for nsSNPs in ACE1. **b** MUpro stability prediction result for MUpro_NN and MUpro_SVM. **c** I-Mutant prediction result for ACE1 protein stability. **d** istable server protein stability prediction using MUpro_SVM, MUpro_NN, iPTREE- STAB and I-Mutant. **Table S4.** Consurf conservation prediction analysis.

**Additional file 2: Fig. S1.** Workflow chart depicting the step-by-step process of insilico analysis of *ACE1* gene. **Fig. S2.** ACE1- Benazepril interactions highlighting interacting residues and interaction types for P351R (**A**), R953Q (**B**), I1018T (**C**), F1051V (**D**), and T1187M (**E**).

## Data Availability

All data generated or analyzed during this study are included in this published article and its supplementary information files. ACE UniProt accession number P1282. Sorting Intolerant from Tolerant (SIFT): https://sift.bii.a-star.edu.sg/. Pro*tein* V*ariation* E*ffect* An*alyzer* (PROVEAN): provean.jcvi.org/seq_submit.php. Polymorphism Phenotyping (PolyPhen-2): http://genetics.bwh.harvard.edu/pph2/. SNPs&GO: https://snps-and-go.biocomp.unibo.it/snps-and-go/. Predictor of human Deleterious Single Nucleotide Polymorphism (PhD-SNPs): https://snps.biofold.org/phd-snp/phd-snp.html. PANTHER: http://www.pantherdb.org/tools/csnpScoreForm.jsp, interpretable decision tree method iPTREE-STAB: http://bioinformatics.myweb.hinet.net/iptree.htm. I-Mutant 2.0: https://folding.biofold.org/i-mutant/i-mutant2.0.html, non-synonymous variations on Protein Stability (INPS): https://inpsmd.biocomp.unibo.it/inpsSuite. Consurf: https://consurf.tau.ac.il/. Robetta: https://robetta.bakerlab.org/. SAVES: https://saves.mbi.ucla.edu/

## Abbreviations

ACE1: Angiotensin-Converting enzyme 1
AA: Amino acid
dbSNPs: Database single-nucleotide polymorphisms
DDG: Delta Delta G
INPS: Impact of non-synonymous variation on protein stability
NCBI: National Centre for Biotechnology Information
nsSNPs: Non-synonymous single nucleotide polymorphisms
PhD-SNP: Predictor of human deleterious single nucleotide polymorphism
Polyphen: Polymorphism phenotyping
PROVEAN: Protein Variation Effect analyzer
SIFT: Sorting Intolerant from Tolerant
SNPs: Single nucleotide polymorphisms
RAS: Renin-angiotensin system
RMSD: Root mean square deviation
