# Supplementary material for: In-silico analysis reveals druggable single nucleotide polymorphisms in angiotensin 1 converting enzyme involved in the onset of blood pressure
Source: BMC Res Notes. 2021 Dec 20;14:457. doi: 10.1186/s13104-021-05879-z (PMC8686250; doi:10.1186/s13104-021-05879-z)
Supplement: Supplementary file 2 — Additional file 2: Fig. S1. Workflow chart depicting the step-by-step process of insilico analysis of ACE1 gene. Fig. S2. ACE1- Benazepril interactions highlighting interacting residues and interaction types for P351R (A), R953Q (B), I1018T (C), F1051V (D), and T1187M (E). [file 13104_2021_5879_MOESM2_ESM.docx]

STRUTURE VERIFICATION

(Tools: Verify-D, ERRAT and PROCHECK)

MOLECULAR DOCKING

(Chimera)

Data retrieval

(dbSNP- NCBI database)

CONSERVATION ANALYSIS

(Consurf)

PROTEIN STABILITY ANALYSIS

(Tools: INSP, MU-PRO, iPSTREE-STAB and I-Mutant)

Deleterious SNPs prediction

(Tools: SIFT, PROVEAN, SNPs&GO, PANTHER, PHD-SNP, Polyphen2)

Supplementary Figure 1: Workflow chart depicting the step-by-step process of insilico analysis of *ACE1* gene.


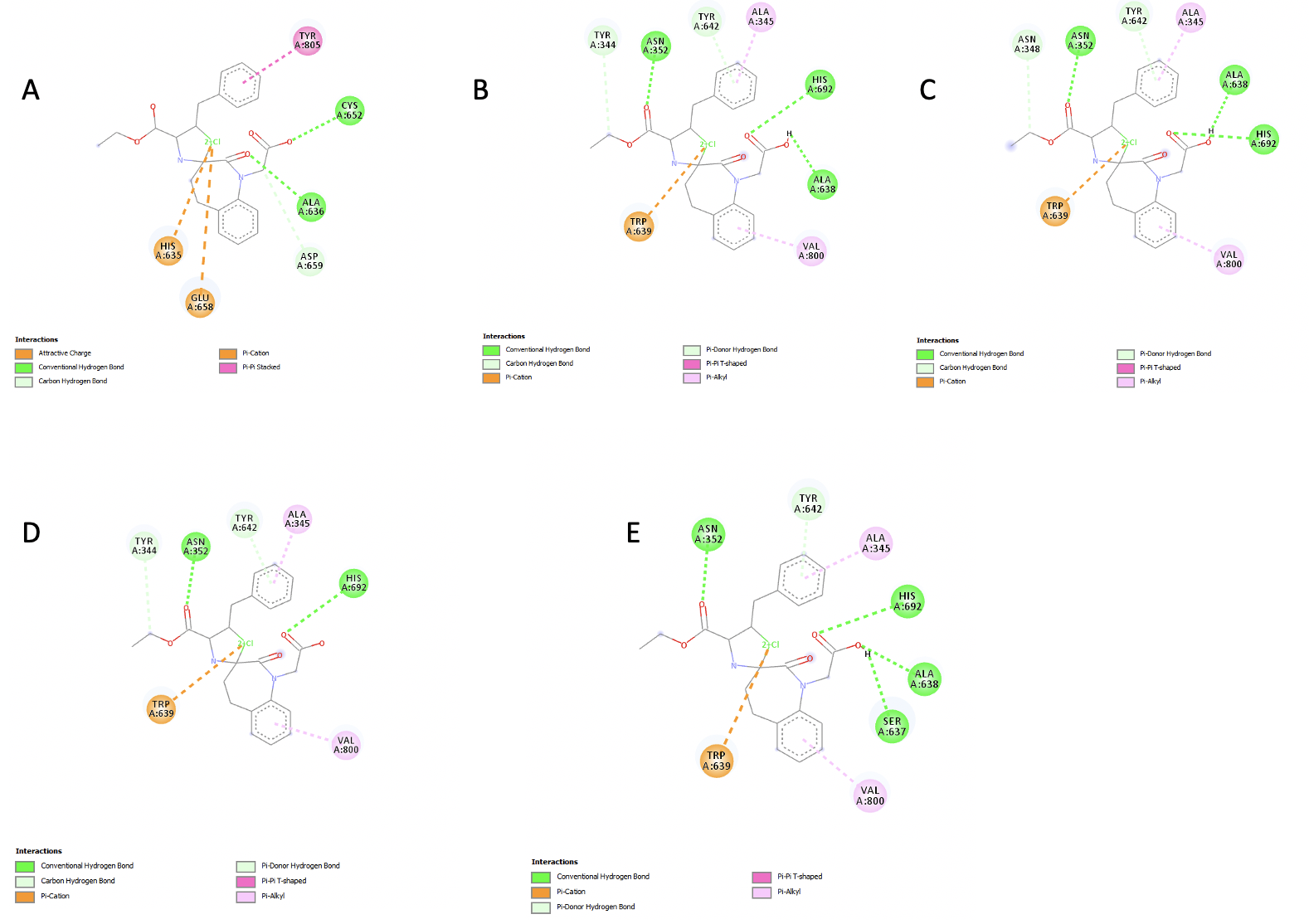


Supplementary Figure 2: ACE- Benazepril interactions highlighting interacting residues and interaction types for P351R (A), R953Q **(B),** I1018T **(C),** F1051V **(D), and** T1187M (E).
